# SMOC2 plays a role in heart failure via regulating TGF-β1/Smad3 pathway-mediated autophagy

**DOI:** 10.1515/med-2023-0752

**Published:** 2023-07-11

**Authors:** Yu Ren, Yun Wu, Wenshuai He, Yingjie Tian, Xingsheng Zhao

**Affiliations:** Scientific Research Department, Inner Mongolia People’s Hospital, Inner Mongolia Autonomous Region, Hohhot, 010017, China; Cardiology Department, Inner Mongolia People’s Hospital, Inner Mongolia Autonomous Region, Hohhot, 010017, China; Clinical Medical Research Center in Cardiovascular Diseases, Inner Mongolia People’s Hospital, Inner Mongolia Autonomous Region, Hohhot, 010017, China

**Keywords:** heart failure, SMOC2, autophagy, TGF-β1/Smad3 signaling pathway

## Abstract

Heart failure (HF) is a major global cause of morbidity and mortality. This study aimed to elucidate the role of secreted protein acidic and rich in cysteine-related modular calcium-binding protein 2 (SMOC2) in HF development and its underlying mechanism. Using a rat HF model, SMOC2 expression was examined and then knocked down via transfection to assess its impact on cardiac function and damage. The study also evaluated the effects of SMOC2 knockdown on autophagy-related molecules and the transforming growth factor beta 1 (TGF-β1)/SMAD family member 3 (Smad3) signaling pathway. Intraperitoneal injection of the TGF-β agonist (SRI-011381) into the HF rat model was performed to explore the SMOC2-TGF-β1/Smad3 pathway relationship. SMOC2 expression was elevated in HF rats, while its downregulation improved cardiac function and damage. SMOC2 knockdown reversed alterations in the LC3-II/I ratio, Beclin-1, and p62 levels in HF rats. Through transmission electron microscope, we observed that SMOC2 knockdown restored autophagosome levels. Furthermore, SMOC2 downregulation inhibited the TGF-β1/Smad3 signaling pathway, which was counteracted by SRI-011381. In conclusion, SMOC2 knockdown inhibits HF development by modulating TGF-β1/Smad3 signaling-mediated autophagy, suggesting its potential as a therapeutic target for HF.

## 1 Introduction

Heart failure (HF) is the terminal stage of most myocardial and vascular diseases such as myocardial infarction and functional heart disorders caused by infections, diabetes, hypertension, or cardio-toxic drugs [1]. It is frequent and has high lethality (5-year mortality rate: over 50%) [2]. Although advanced interventions, such as cardiac transplantation, cardiac resynchronization therapy, and pharmacological treatment, have been used to control HF-related symptoms [3,4], the mortality of HF remains high. Therefore, it is still imperative to further elucidate key mechanisms underlying HF, which may help to develop novel therapeutic strategies to decrease the mortality of HF patients.

Secreted protein acidic and rich in cysteine (SPARC)-related modular calcium-binding protein 2 (SMOC2) belongs to the SPARC family and is expressed in many tissues [5,6]. SMOC2 has been reported to be implicated in diverse cellular functions such as angiogenesis [7] and metastasis [8]. Accumulating evidence has revealed that SMOC2 participates in a variety of diseases, including asthma [9], cancers [10,11], and atrial fibrillation [12]. Notably, SMOC2 is found to be differentially expressed in failing right ventricular and may be a potential target for HF [13]. Laugier et al. also found that SMOC2 was implicated in matrix remodeling and associated with fibrosis in chronic Chagas disease cardiomyopathy, a disease presenting with HF and arrhythmia [14]. These data suggest a potential role of SMOC2 in HF. Despite these, the function and regulatory mechanism of SMOC2 in HF have not been completely elucidated.

Transforming growth factor beta 1 (TGF-β1) is a pro-fibrotic cytokine, which has significant implications for wound repair and regeneration [15]. SMAD family member 3 (Smad3) is a well-recognized protein that acts as a switch in TGF-β1 signal transduction [16]. In recent years, the TGF-β1/Smad3 signaling pathway has been found to play a crucial role in cardiac fibrosis [17,18]. An animal study indicated that TGF-β1 and phosphorylated Smad3 were elevated in myocardial infarction rats and blocking the TGF-β1/Smad3 axis could restore cardiac function and mitigate myocardial fibrosis [19]. Several studies have revealed that TGF-β1/Smad3 signaling pathway participates in the development of HF [20,21]. However, whether SMOC2 contributes to the development of HF via targeting this pathway remains largely unknown.

In this study, we established a rat model of HF and investigated the effects of SMOC2 knockdown on cardiac function and damage in the HF rat model. Moreover, we explored the effects of SMOC2 on autophagy and the regulatory mechanism of TGF-β1/Smad3 axis on autophagy in HF. Our study aimed to elucidate the role of SMOC2 in the onset and development of HF and its underlying mechanism, thus providing a novel target for the prevention and treatment of HF.

## 2 Materials and methods

### 2.1 Animals

Thirty male Sprague-Dawley rats (8 weeks old, 230 ± 15 g) were used in this study, which were obtained from GemPharmatech Co. Ltd (Nanjing, China). All the rats were bred in cages (3 rats/cage) under pathogen-free conditions with a temperature of 23 ± 1°C, a humidity of 55 ± 5%, and a 12 h light/dark cycle. The rats were provided with free access to water and food.

### 2.2 Construction of recombinant lentiviral vectors

The short hairpin RNA of SMOC2 (sh-SMOC2) sequence (GCATGGACATTGACCCTTATA) was inserted into pGCSIL-GFP (GeneChem) lentiviral plasmids. The 293T cells were co-transfected with the lentiviral plasmid carrying sh-SMOC2, packaging vector psPAX2, and envelope vector pMD2.G using HighGene transfection reagent (Abclonal, Boston, MA, USA) to obtain the recombinant lentivirus. At 48 h of post-transfection, viral supernatants were harvested. By further passing through 0.45 µm filters (Millipore, Bedford, MA, USA) the virus was concentrated with PEG-8000. The next day, the viral pellet was collected, re-suspended, and frozen at −80°C until use.

### 2.3 Establishment of HF rat model

To establish the rat models of HF, rats were adaptively fed for 1 week and fasted for 12 h. The rats were intraperitoneally injected with 1.5% sodium pentobarbital (50 mg/kg) for anesthesia. Then, the left coronary artery of rats was ligated at the position between the left atrial appendage and the pulmonary conus on a temperature-controlled heating pad. The abdomen was sutured in layers. Rats in the sham operation group underwent the same procedure but without the left coronary artery ligation. To further investigate the function of SMOC2 in HF development, lentivirus carrying sh-SMOC2 or short hairpin RNA negative control (sh-NC) was injected into the infarct area of HF rats immediately after induction of myocardial infarction. Therefore, rats were randomly assigned into four groups (*n* = 6/group): sham operation (sham), HF rat model (HF), HF rat model treated with lentivirus carrying sh-NC (HF + sh-NC), and HF rat model treated with lentivirus carrying sh-SMOC2 (HF + sh-SMOC2).

To explore the relationship between SMOC2 and TGF-β1/Smad3 signaling pathway, TGF-β agonist (SRI-011381, 30 mg/kg daily) was intraperitoneally injected into rat model 5 days after lentivirus injection. After 4 weeks of injection, rats were sacrificed by cervical dislocation, and myocardial tissues were collected for subsequent assays.

### 2.4 Quantitative PCR (qPCR)

Total RNA was extracted from myocardial tissues using Trizol reagent (Invitrogen, USA). Reverse transcription for the synthesis of cDNA was performed using PrimeScriptTM RT Master Mix Kit (Takara, Japan). Real-time qPCR was conducted to detect SMOC2, TGF-β1, and Smad3 expression using the SYBR Green PCR Master Mix kit (Lifeint, Xiamen, China) in a Stratagene Mx3000P PCR machine (Agilent, California, CA, USA). The primer sequences are listed in Table 1. Glyceraldehyde 3-phosphate dehydrogenase (GAPDH) was used as the internal reference. Relative gene expression was calculated using the 2^−ΔΔCT^ method.

**Table 1:** Primers used for qRT-PCR

Genes	Primer sequences (5′-3′)	
	Forward	Reverse
SMOC2	GGAAAAGCAGTCTCCTTGCAAAT	TGGGCTGCTCATACCTTGTG
Acta2	AGACCCTCTTCCAGCCATCT	CCCCGAGAGGACGTTGTTAG
TGF-β1	TGGCCAGATCCTGTCCAAAC	AGAAGTTGGCATGGTAGCCC
Smad3	AGAAGCTCAAGAAGACGGGG	TGAAGCGTGGAATGTCTCCC
GAPDH	TGTGGGCATCAATGGATTTGG	ACACCATGTATTCCGGGTCAAT

### 2.5 Western blot

Total proteins were extracted from myocardial tissues using radio immunoprecipitation assay lysis buffer (Beyotime, Haimen, China) containing phenylmethanesulfonyl fluoride at 4°C for 15 min. After determining the protein concentration using bicinchoninic acid Protein Assay kit (Solarbio, Beijing, China), protein extracts (50–100 μg) were electrophoresed on 10% sodium dodecyl sulfate polyacrylamide gel electrophoresis and transferred onto polyvinylidene fluoride membranes (Millipore). After blocking, membranes were incubated with primary antibodies to SMOC2 (0.4 μg/mL, ab121675; Abcam, Cambridge, UK), LC3II/I (1:1,000, 12741; Cell Signaling Technology, CST, MA, USA), Beclin-1 (1:1,000, ab207612; Abcam), p62 (1:1,000, 23214; CST), Smad3 (1:1,000, ab40854; Abcam), p-Smad3 (1:1,000, 9520; CST), TGF-β1 (1:1,000, ab215715; Abcam), and GAPDH (1:10,000, ab181602; Abcam) overnight at 4℃. Subsequently, the membranes were probed with corresponding goat anti-rabbit IgG H&L (HRP) secondary antibodies (1:2,000, ab205718; Abcam) for 60 min away from light. Protein blots were visualized by incubation with a chromogenic substrate using the enhanced chemiluminescence method. The gray value of protein bands was analyzed using Image J software (version 1.37).

### 2.6 Echocardiographic measurements

Cardiac function after surgery was assessed by echocardiography using VisualsonicVevo 2100 imaging system. Before evaluation, rats were anesthetized with isoflurane at 2.5 L/min. Vevo Vasc Analysis Software was then used to analyze left ventricular end systolic diameter (LVESD), left ventricular end diastolic diameter (LVEDD), left ventricular ejection fraction (EF), and fractional shortening (FS).

### 2.7 Hematoxylin and eosin (HE) staining

Myocardial tissues were fixed in 4% paraformaldehyde at 4°C for 24 h. Then, the tissues were dehydrated with gradient ethanol (50, 75, 85, 90, and 100%), followed by soaking in xylene for 30 min. Subsequently, the tissues were embedded in paraffin and sectioned to 4–7 μm. Afterward, paraffin sections were deparaffinized and stained with hematoxylin for 5 min and 0.5% alcohol-eosin for 1–2 min. After dehydration with absolute ethanol and clearing in xylene, the sections were observed under a microscope (BX53, Olympus, Tokyo, Japan).

### 2.8 Masson staining

Paraffin sections of myocardial tissues were dewaxed and hydrated. Then, the sections were stained with Weigert’s iron hematoxylin for 10 min, followed by being differentiated with acidic ethanol solution. Afterward, the sections were stained with Masson blue solution, washed with distilled water, and stained with Lichun Red magenta solution for 10 min. Then sections were treated with the weak acid working solution (distilled water: weak acid solution = 2:1) for 1 min, differentiated in phosphomolybdic acid solution for 1 min, immersed in aniline blue for 1 min, and rapidly dehydrated in 95% ethanol. The sections were permeabilized in xylene three times with each for 10 s. Finally, the sections were sealed with neutral resin and counted under a microscope (BX53, Olympus), and the interstitial collagen volume fraction (CVF) was quantified by Image Pro Plus software.

### 2.9 Enzyme-linked immunosorbent assay (ELISA)

According to the manufacturers’ directions, ELISA kits were used to assess the levels of N-terminal proBNP (NT-proBNP), cardiac troponin (cTnI), and cardiac troponin T (cTnT) in the serum of rats. The optical density was assessed at 450 nm under a microplate reader (DR-3518G, Hiwell Diatek, Wuxi, China) and quantitative analyses were conducted using ImageJ software. The following ELISA kits were used in this study: NT-proBNP (#E-EL-R3023, Elabscience, Wuhan, China), cTnI (#E-EL-R3023, Elabscience), and cTnT (#E-EL-R3023, Elabscience).

### 2.10 Transmission electron microscope (TEM)

The state of autophagy in rat myocardial tissues was observed under TEM. In brief, myocardial tissues were fixed with 2.5% glutaraldehyde at 25°C. Then, the tissues were dehydrated with graded acetone (50, 70, 90, and 100%) and embedded in Epon 812 resin (Head, Beijing, China) overnight. Ultrathin sections (50–70 nm) were acquired using an EM UC7 ultramicrotome (Leica, Germany) and then stained with 2% sodium acetate–lead citrate. Finally, the sections were observed under an FEI Tecnai TEM (Thermo Fisher Scientific, Waltham, MA, USA).

### 2.11 Statistical analysis

All data are expressed as mean  ±  standard deviation. Using one-way analysis of variance, statistical differences between groups were compared. All statistical analyses were performed with GraphPad 7.0 software, and *P* < 0.05 was considered statistically significant.


**Ethical approval:** This work was conducted in accordance with the Declaration of Helsinki (1964). All animal experimental procedures were approved by the Animal Ethics Committee of Inner Mongolia People’s Hospital. This study conformed to the guidelines for the Care and Use of Laboratory Animals (National Institutes of Health, US, 8th Edition, 2011).

## 3 Results

### 3.1 SMOC2 knockdown ameliorated pathological injury of HF

**Figure 1:**
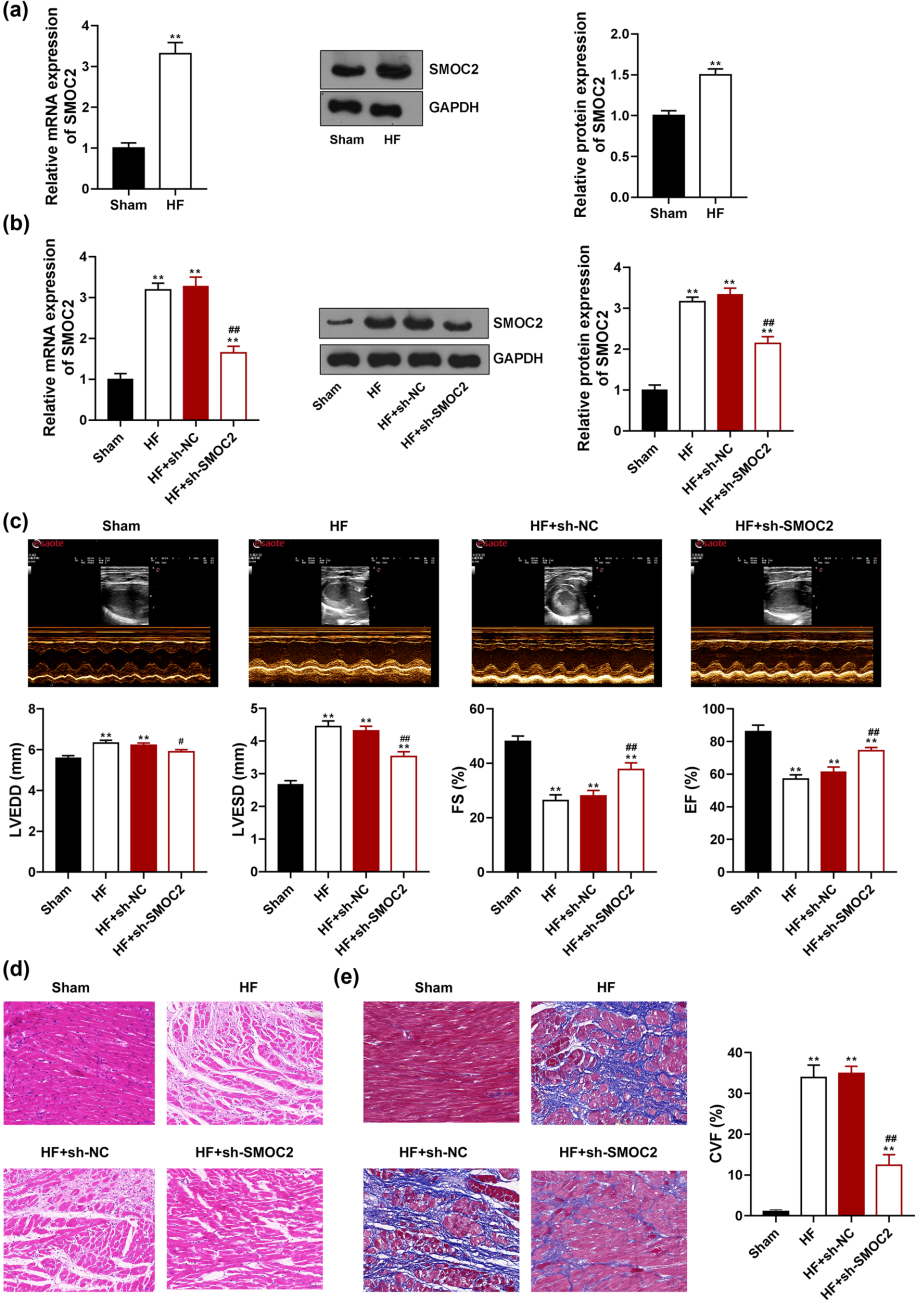
Downregulation of SMOC2 improved cardiac function and damage in HF rats. (a) qPCR and western blot assays showed SMOC2 expression in HF rats. (b) qPCR and western blot assays showed SMOC2 expression in HF rats after SMOC2 knockdown. (c) Echocardiographic parameters of HF rats after SMOC2 knockdown. LVESD: left ventricular end-systolic diameter; LVEDD: left ventricular end-diastolic diameter; EF: left ventricular ejection fraction; FS: left ventricular fractional shortening. (d) and (e) HE and Masson staining showed pathological changes in myocardial tissues of HF rats after SMOC2 knockdown (scale bar = 50 µm). CVF: collagen volume fraction. **P < 0.01 compared to sham group; ^#^ *P* < 0.05 and ^##^ *P* < 0.01 compared to HF + sh-NC group.

To investigate the role of SMOC2 in HF, we first determined SMOC2 expression in the HF rat model. Results showed that compared to sham rats, both SMOC2 mRNA and protein expression was dramatically increased in the myocardial tissues of HF rats (*P* < 0.01, Figure 1a). We further knocked down SMOC2 expression in the HF rat model. Results showed that compared to HF + sh-NC group, both SMOC2 mRNA and protein expression levels were distinctly decreased in HF + sh-SMOC2 group (*P* < 0.01, Figure 1b), manifesting that SMOC2 was successfully knocked down in the HF rat model. Echocardiographic measurements revealed that LVESD and LVEDD were dramatically increased and EF and FS were significantly decreased in the HF group in comparison with those in the sham group (*P* < 0.01, Figure 1c), confirming that the HF rat model was successfully established. As expected, knockdown of SMOC2 remarkably decreased the values of LVESD and LVEDD and increased EF and FS of HF rats (*P* < 0.05, Figure 1c), indicating that downregulation of SMOC2 improved cardiac function in HF. Moreover, HE staining showed that the myocardial fibers were arranged neatly, and the nuclei and cytoplasm were uniformly colored in the sham group; myocardial cells were obviously sparse and hypertrophic, and the myocardial fibers were thickened and spaced in HF rats. SMOC2 knockdown mitigated the pathological damage in the myocardial tissues of HF rats (Figure 1d). Similar results were obtained from Masson staining that blue-stained collagen fibers were occasionally observed in the myocardial fibers of the sham group, but more in the myocardial fibers of HF rats (*P* < 0.01, Figure 1e). The blue-stained collagen fibers in HF rats were significantly decreased after SMOC2 knockdown (*P* < 0.01, Figure 1e).

**Figure 2:**
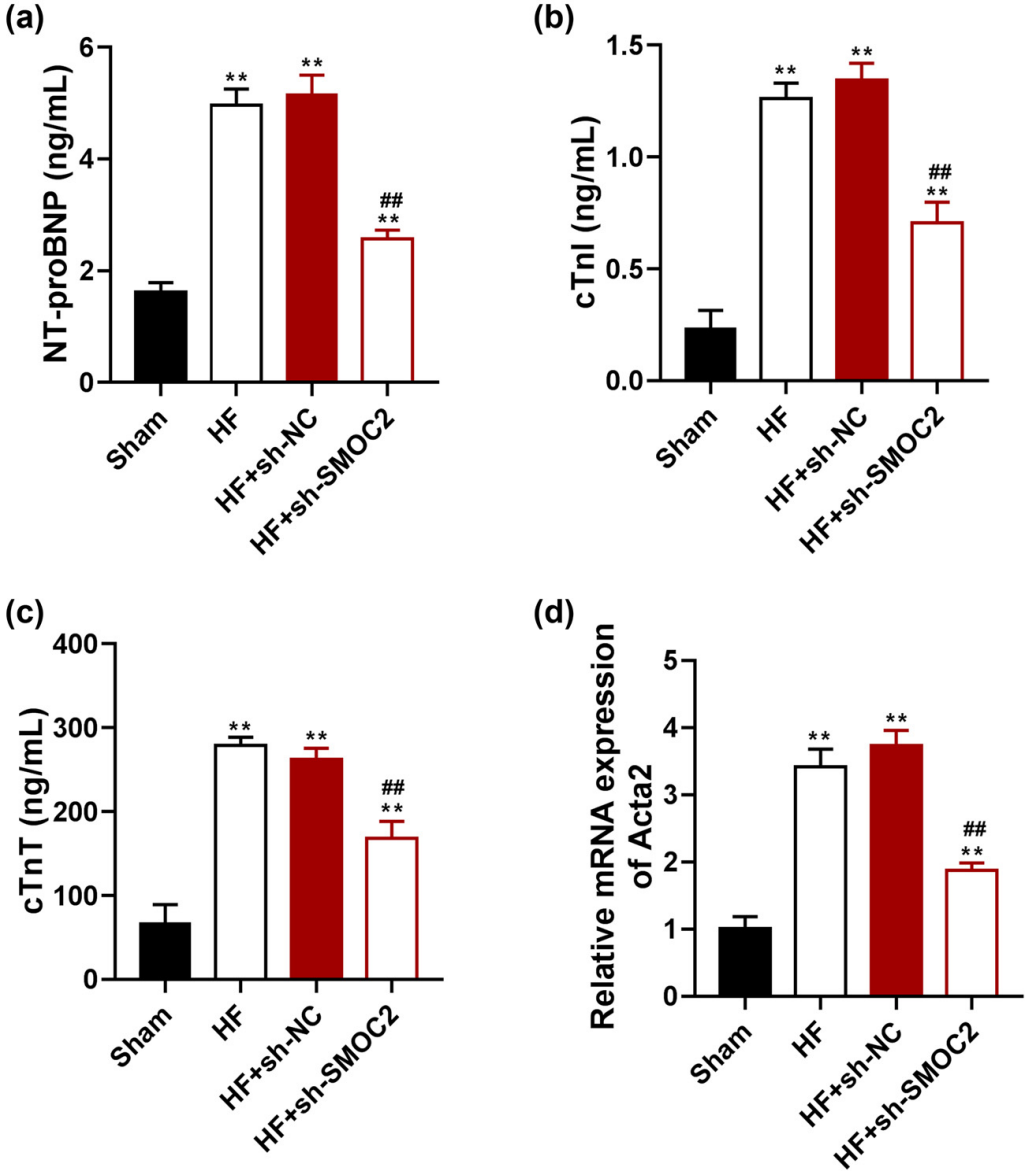
Downregulation of SMOC2 alleviated myocardial injury in HF rats. (a)–(c) ELISA showed the levels of serum NT-proBNP, cTnI, and cTnT in HF rats after SMOC2 knockdown. (d) qPCR showed Acta2 expression in HF rats after SMOC2 knockdown. **P < 0.01 compared to sham group; ^##^ *P* < 0.01 compared to HF + sh-NC group.

To further determine the alleviating effect of SMOC2 knockdown in the myocardial injury of HF, the levels of the common myocardial injury markers, NT-proBNP, cTnI, and cTnT were evaluated. ELISA results showed that the levels of NT-proBNP, cTnI, and cTnT in HF rats were markedly higher than those in sham rats (*P* < 0.01), but these levels were decreased in HF rats following SMOC2 knockdown (*P* < 0.01; Figure 2a–c). Moreover, we observed that the mRNA expression of fibrosis marker Acta2 was significantly increased in the myocardial tissues of HF rats compared with that of sham rats (*P* < 0.01), which was reversed after SMOC2 knockdown (*P* < 0.01; Figure 2d).

### 3.2 Downregulation of SMOC2 regulated autophagy in HF

**Figure 3:**
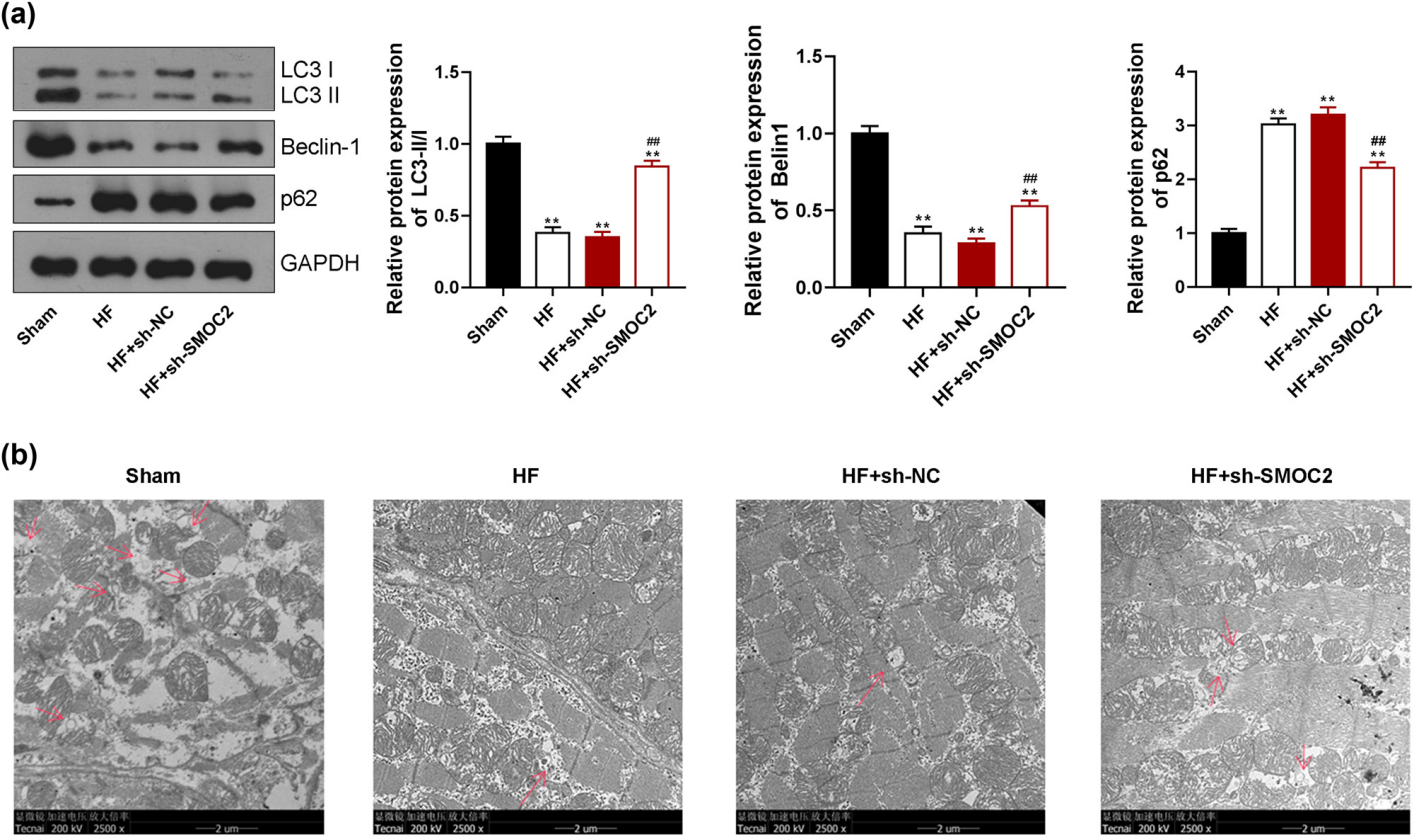
Downregulation of SMOC2 regulated autophagy in HF rats. (a) Western blotting showed the expression of autophagy-related markers (LC3 II/I, Beclin-1, and p62) in HF rats after SMOC2 knockdown. (b) TEM showed the state of autophagy in HF rats after SMOC2 knockdown (scale bar = 2 µm). **P < 0.01 compared to sham group; ^##^ *P* < 0.01 compared to HF + sh-NC group.

To elucidate the possible mechanism of SMOC2 in HF development, we explored the effect of SMOC2 knockdown on autophagy-related proteins. We found that the LC3-II/I ratio and Beclin-1 expression were visibly decreased, and p62 expression was increased in HF group compared with sham group (*P* < 0.01). SMOC2 knockdown increased the levels of LC3-II/I and Beclin-1 and reduced the p62 level in HF rats (*P* < 0.01, Figure 3a). Furthermore, we observed that decreased autophagosomes in the myocardial tissues of HF rats under TEM, which was reversed following SMOC2 knockdown (Figure 3b).

### 3.3 Downregulation of SMOC2 inhibited TGF-β1/Smad3 signaling pathway in HF

**Figure 4:**
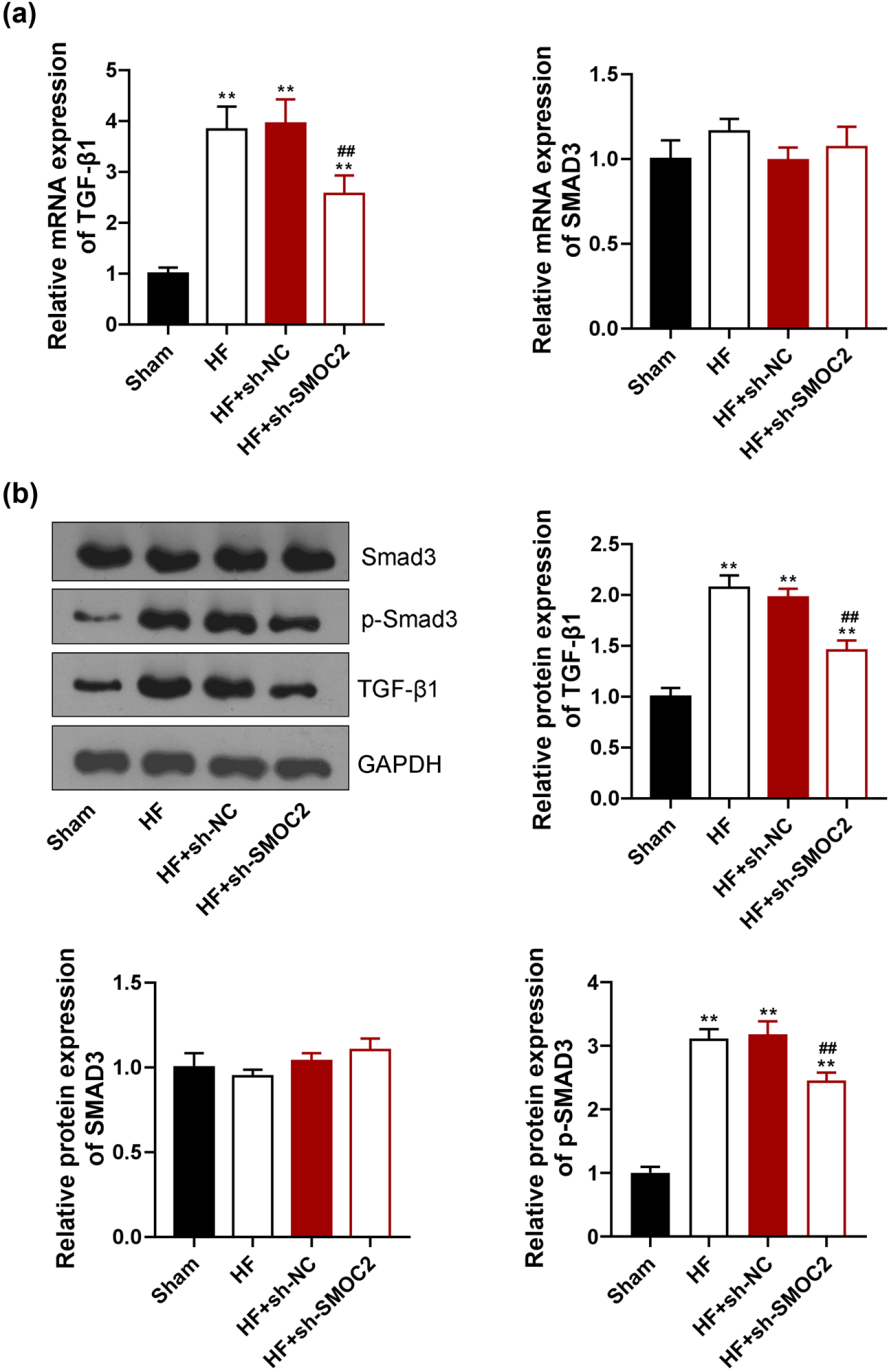
Downregulation of SMOC2 inhibiting TGF-β1/Smad3 signaling pathway. (a) qPCR showed TGF-β1 and Smad3 expression in HF rats after SMOC2 knockdown. (b) Western blot assay showed TGF-β1, Smad3, and p-Smad3 expression in HF rats after SMOC2 knockdown. **P < 0.01 compared to sham group; ^##^ *P* < 0.01 compared to HF + sh-NC group.

The effects of SMOC2 knockdown on TGF-β1/Smad3 signaling pathway in HF were investigated. qPCR and western blot displayed that TGF-β1 expression was visibly upregulated in HF group relative to sham group (*P* < 0.01). SMOC2 knockdown downregulated the expression of TGF-β1 in the HF rats (*P* < 0.01, Figure 4a and b). Meanwhile, western blot demonstrated that p-Smad3 expression was dramatically increased in HF group, which was remarkably decreased after knockdown of SMOC2 (*P* < 0.01, Figure 4b).

### 3.4 Downregulation of SMOC2 alleviated myocardial damage and regulated autophagy via inhibiting the TGF-β1/Smad3 pathway in HF

**Figure 5:**
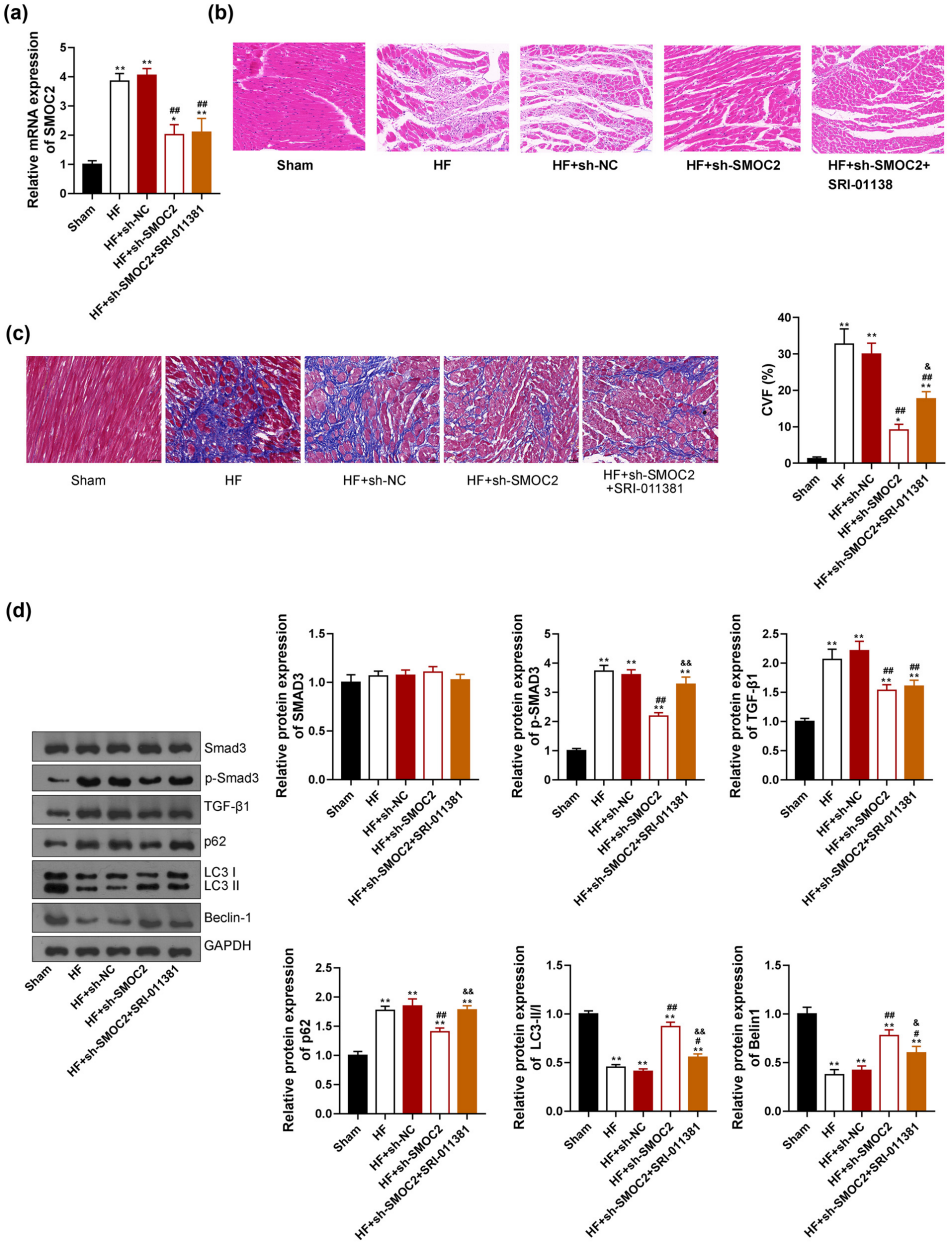
Downregulation of SMOC2 affected autophagy and mitigated cardiac damage in HF rats via inhibiting the TGF-β1/Smad3 signaling pathway. TGF-β agonist (SRI-011381, 30 mg/kg daily) was intraperitoneally injected into the HF rats that were transfected with sh-SMOC2. (a) qPCR showed SMOC2 expression in rats. (b) and (c) HE and Masson staining showed pathological changes in the myocardial tissues of rats (scale bar = 50 µm). CVF: collagen volume fraction. (d) Western blot assay showed the expression of autophagy- and TGF-β1/Smad3 signaling pathway-related proteins in rats. *P < 0.05 and **P < 0.01 compared to sham group; ^#^ *P* < 0.05 and ^##^ *P* < 0.01 compared to HF + sh-NC group; ^&^ *P* < 0.05 and ^&&^ *P* < 0.01 compared to HF + sh-SMOC2 group.

To further investigate whether SMOC2 regulates autophagy and cardiac function in HF via the TGF-β1/Smad3 pathway, TGF-β agonist (SRI-011381) was intraperitoneally injected into rat model 5 days after lentivirus injection. Results showed that SMOC2 expression had no significant difference between HF + sh-SMOC2 and HF + sh-SMOC2 + SRI-011381groups (Figure 5a). Moreover, HE staining revealed that cardiac damage degree of HF + sh-SMOC2 group was lighter than that of HF + sh-SMOC2 + SRI-011381 group, suggesting that SRI-011381 inhibited the alleviating effect of SMOC2 knockdown on cardiac damage (Figure 5b). Masson staining also showed that the blue-stained collagen fibers in HF + sh-SMOC2 + SRI-01138 group were significantly more than 1 HF + sh-SMOC2 group (*P* < 0.01, Figure 5c), confirming that SRI-011381 inhibited the effect of SMOC2 knockdown in HF rats. Furthermore, western blotting showed that SRI-011381 treatment reversed the effects of sh-SMOC2 on decreasing the levels of p-Smad3 and p62 and increasing LC3-II/I ratio and Beclin-1 levels in HF rats (*P* < 0.05, Figure 5d).

## 4 Discussion

HF has been a serious threat to human health with high morbidity and mortality worldwide. Early detection of HF is crucial for managing this disease [22], and a better understanding of the molecular mechanism underlying HF could help clinical treatment [23]. A previous study used multiple feature selection strategies to compare the gene expression profiles between patients with HF and without HF and revealed that SMOC2 might play a vital part in HF development [24]. Noteworthily, SMOC2 was found to be highly expressed in patients with HF compared with those without HF [24]. However, the mechanism underlying SMOC2 mediating HF development is largely unknown. Herein, we investigated the regulatory role of SMOC2 and its mechanism in HF development. Consistent with the previous research, our results indicated that SMOC2 expression was increased in the HF rat model, and downregulation of SMOC2 improved cardiac function.

Myocardial fibrosis is one of the primary causes of HF [25–27], mainly characterized by imbalanced synthesis and degradation of extracellular matrix, as well as imbalanced and disorderly arrangement of diverse types of collagen [28]. A bioinformatic analysis-based study identified SMOC2 as a promising non-invasive biomarker of kidney fibrosis [29]. Moreover, it has been reported that SMOC2 silencing can suppress pulmonary fibrosis [30] and kidney fibrosis [31]. In the present study, we found that SMOC2 knockdown effectively inhibited myocardial fibrosis in HF rats and alleviated myocardial injury, which was consistent with the previous studies [30,31].

Autophagy is a cellular self-renewal process, which depends on the degradation of the cytoplasmic proteins or organelles in lysosomes [32]. Beclin-1, LC3, and p62 are main factors involved in the regulation of autophagy. The formation of the phagophore leads to LC3-I lipidation, converting it to LC3-II [33]. Thus, the LC3-II/LC3-I ratio serves as a key indicator of autophagy induction, estimating the functional status of autophagy [33]. p62 is a versatile scaffold protein with multiple domains, which interacts with ubiquitinated proteins and facilitates their degradation through the proteasome, and it can also bind directly to LC3 to deliver ubiquitinated proteins for degradation by autophagy [33]. Autophagy can be activated by various stress conditions, like inflammation, ischemia and reperfusion, oxidative stress, metabolic stress, and energy crisis [34,35]. Mild-to-moderate autophagy induction is considered to be cytoprotective, while excessive or uninhibited autophagy induction may be detrimental [36]. Increasing studies have reported that autophagy plays a significant role in modulating cardiovascular diseases [37,38]. Autophagy is also shown to be activated in cardiomyocytes of HF patients [39] and implicated in the pathophysiology of HF [36]. Wang et al. demonstrated that the extract of *Panax notoginseng*, named Panax Notoginseng saponins, exerted a cardioprotective effect in HF via inducing autophagy [40]. Another animal study demonstrated that rapamycin could ameliorate cardiac function in chronic HF rats by enhancing autophagy [41]. These investigations indicate the complexity of the autophagy mechanism in HF. On the other hand, SMOC2 plays a pro-fibrotic role in the mouse model of chronic kidney disease via autophagy activation [42], suggesting a potential association between SMOC2 and autophagy. Nevertheless, little is known about the correlation between SMOC2 and autophagy in the onset and development of HF. In this study, the decreased LC3-II/I ratio and Beclin-1 expression, autophagosomes, and augmented p62 expression in HF rats were significantly reversed after SMOC2 knockdown, implying that SMOC2 knockdown might inhibit HF via regulating autophagy.

TGF-βs are multifunctional growth factors involved in the regulation of key processes of development, disease, and tissue repair [43]. As a pivotal member of the TGF-β superfamily, TGF-β1 is regarded as the core mediator in fibrogenesis, which is activated and elevated in fibrotic disorders [44]. Significantly, TGF-β1 activation has been reported to lead to myocardial fibrosis by affecting the deposition of extracellular matrix that is a contributing factor in HF pathogenesis [45,46]. Smad3 is the key downstream mediator of the TGF-β1 signaling pathway in myocardial fibrosis [47]. Increasing evidence reveals that the TGF-β1/Smad3 signaling pathway has significant implications in HF development [20,21]. Relevant data have indicated that suppression of the TGF-β1/Smad3 signaling pathway could attenuate myocardial fibrosis to prevent the progression and development of HF [28,48]. Notably, SMOC2 deficiency was found to reduce bleomycin-induced pulmonary fibrosis by restraining TGF-β1/Smads pathway [30]. Furthermore, accumulating data have demonstrated the crucial role of the TGF-β1/Smad3 pathway in autophagy [49,50]. A previous investigation suggested that inhibition of the TGF-β1/Smad3 signal transduction cascade could ameliorate liver fibrosis by suppressing autophagy [51]. Nonetheless, a recent study has indicated that blocking the TGF-β1/Smad3/ERK/P38 signaling pathway can enhance autophagy to mitigate pulmonary fibrosis [52]. Herein, we found that downregulation of SMOC2 inhibited TGF-β1/Smad3 signaling pathway, which conformed to the previous research [30]. Moreover, SRI-011381 (a TGF-β agonist) reversed the anti-fibrotic and pro-autophagic effects of SMOC2 knockdown on myocardial tissues of HF rats. Therefore, we speculate that TGF-β1/Smad3 signaling pathway is a key mechanism to mediate the role of SMOC2 in HF development.

In conclusion, our findings reveal that SMOC2 was upregulated in HF rats. SMOC2 knockdown improved cardiac function and alleviated pathological injury in HF rats by regulating autophagy via TGF-β1/Smad3 signaling pathway blockage. SMOC2 may serve as a potential target for HF treatment.
